# Clinical outcomes of second-generation limus-eluting stents compared to paclitaxel-eluting stents for acute myocardial infarction with cardiogenic shock

**DOI:** 10.1371/journal.pone.0214417

**Published:** 2019-04-03

**Authors:** Chun-Tai Mao, Tien-Hsing Chen, Chi-Nan Tseng, Shao-Wei Chen, I-Chang Hsieh, Ming-Jui Hung, Pao-Hsien Chu, Chao-Hung Wang, Ming-Shien Wen, Wen-Jin Cherng, Dong-Yi Chen

**Affiliations:** 1 Division of Cardiology, Department of Internal Medicine, Chang Gung Memorial Hospital, Keelung, Taiwan; 2 Department of Thoracic and Cardiovascular Surgery, Chang Gung Memorial Hospital at Linkou, Taoyuan, Taiwan; 3 Division of Cardiology, Department of Internal Medicine, Chang Gung Memorial Hospital at Linkou, Taoyuan, Taiwan; CVPath Institute Inc., University of Maryland, UNITED STATES

## Abstract

**Objective:**

Whether the cardiovascular (CV) outcomes of second-generation limus-eluting stents (LESs) differ from those of paclitaxel-eluting stents (PESs) in patients with acute myocardial infarction (AMI) complicated by cardiogenic shock (CS) is still unclear.

**Methods:**

We used the Taiwan National Health Insurance Research Database to analyse data of 516 patients with AMI and CS diagnosed from January 2007 to December 2011. We used propensity score matching to adjust for the imbalance in covariate baseline values between these two groups. We evaluated clinical outcomes by comparing 197 subjects who used second-generation LESs to 319 matched subjects who used PESs.

**Results:**

The risk of the primary composite outcomes (i.e., myocardial infarction, coronary revascularisation or CV death) was significantly lower in the second-generation LES group than in the PES group [37.3% vs. 51.8%; hazard ratio (HR), 0.73; 95% CI: 0.56–0.95] at the 12-month follow-up. The patients who received second-generation LESs had a lower risk of coronary revascularisation (HR 0.62; 95% CI: 0.41–0.93) than those who used PESs. However, the risks of myocardial infarction (HR 0.56; 95% CI: 0.26–1.24), ischemic stroke (HR 0.73; 95% CI: 0.23–2.35), or CV death (HR 0.90; 95% CI: 0.63–1.28) were not significantly different between the two groups.

**Conclusions:**

Among patients with CS-complicating AMI, second-generation LES implantation significantly reduced the risk of coronary revascularisation and composite CV events compared to PES implantation at the 12-month follow-up.

## Introduction

Cardiogenic shock (CS) is the leading cause of death associated with acute myocardial infarction (AMI), with an incidence of approximately 5%–15% [1]. Revascularisation strategies have been recommended for patients with AMI with CS, hoping to improve the high morbidity and mortality rates in this group of patients. Early revascularisation therapy (percutaneous coronary intervention [PCI] or coronary artery bypass graft surgery), when compared with initial medical stabilisation, has been shown to improve the long-term survival of the CS-AMI patients [2]. However, the choice of an appropriate coronary stent during PCI for patients with AMI and CS remains debatable [3]. Drug-eluting stents (DESs) have been reported to be more efficient than bare-metal stents (BMSs) for reducing mortality or repeat revascularisation rates among AMI patients and even in CS-AMI patients [4–7]. However, there is not enough evidence to determine which type of DESs is associated with better clinical outcomes in CS-AMI patients. Second-generation limus-eluting stent (LES) implantation has been reported to have better outcomes than paclitaxel-eluting stent (PES) implantation in terms of stent thrombosis rates, target lesion revascularisation or major adverse cardiac events [8–11]. However, most of these studies excluded patients with AMI and CS.

Owing to the limited data available regarding the cardiovascular (CV) outcomes of different DES types in CS-AMI patients, we used data from the Taiwan National Health Insurance Research Database (NHIRD) to conduct a nationwide cohort study on patients with AMI and CS, comparing the clinical outcomes of second-generation LESs versus those of PESs as reflected by the CV outcomes, including myocardial infarction (MI), ischemic stroke (IS), coronary revascularisation and CV mortality.

## Materials and methods

### Data source

For this study, we analysed data from the NHIRD released by the Taiwan National Health Research Institute. The NHIRD comprises healthcare data of 99.9% of the Taiwanese population including data on date of birth, gender, diagnostic codes (International Classification of Diseases, Ninth Revision, Clinical Modification [ICD-9-CM] codes), surgical procedures, drug prescriptions, hospitalisations and expenditure amounts. We made sure to de-identify the information and records of patients prior to analysis to ensure patient anonymity. The Ethics Institutional Review Board of Chang Gung Memorial Hospital approved this study.

### Study cohort identification

We identified patients with AMI (ICD-9-CM code 410) and CS who received DES implantations by PCIs during the period from January 2007 to December 2011. The patients who need inotropic agents or intra-aortic pump (IABP) to keep stable hemodynamics are considered in the status of CS.[6, 12] We define CS as: (1) the need for dopamine doses >1320 mg; (2) the need for norepinephrine >132 mg; (3) the need of IABP; or (4) the need of epinephrine 1mg at least twice for resuscitation. In the study of Intra-aortic Balloon Support for Myocardial Infarction with Cardiogenic Shock (IABP-SHOCK II), the median dosage of dopamine was approximately 4.1–4.2 μg/kg per minute and 0.3–0.4 μg/kg per minute of norepinephrine[13]. According to recent American Heart Association guidelines, 5 μg/kg per minute of dopamine or 0.5 μg/kg per minute of norepinephrine are the reasonable dosage of inotropic agents to stabilize the hemodynamic status[14, 15]. The median duration of catecholamines in IABP-SHOCK II study was 3 days [13]. Therefore, we defined the amount of catecholamine dose of dopamine >1320 mg or norepinephrine >132 mg which are approximately 5 μg/kg per minute for a 60 kg adult for 3 days (dopamine) and 0.5 μg/kg per minute for a 60 kg adult for 3 days (norepinephrine).

The data quality and major diseases such as AMI, ischemic stroke, diabetes or chronic kidney disease (CKD) in the claims database had been validated [16–19]. We defined the index date as the admission date for AMI, and we classified patients into second-generation LES or PES groups according to the type of stent they received. We classified patients using everolimus, zotarolimus or biolimus into the second-generation LES group, and those receiving paclitaxel stents as the PES group. S1 Fig shows the flowchart of the study cohort enrolment. We analysed data from all patient follow-ups up until their date of death, withdrawal from the NHI or December 31, 2011, whichever occurred first.

### Comorbidity and study outcomes

We used the ICD-9-CM diagnostic codes (S1 Table) to define comorbidities. The primary outcomes included the composite CV events of MI, coronary revascularisation and CV death. We defined MIs as admissions with a diagnosis of acute MI validated in the previous NHIRD studies [18]. We used the Taiwan’s NHI procedure codes to detect coronary revascularisation. We defined CV deaths according to the criteria of the Standardised Definitions for End Point Events in Cardiovascular Trials, published by the Food and Drug Administration [20]. The definition of CV death has been published [5, 6]. Deaths were identified as NHI programme withdrawals [21]. The secondary outcomes of interest included death from any cause, admission due to heart failure and stroke. The diagnostic codes of heart failure and stroke were also validated.

### Statistical analysis

To compare the two study groups on clinical outcomes, we conducted a propensity score matching (PSM) to balance the distribution of baseline characteristics between groups. We matched each patient in the PES group with two patients (if possible) in the second-generation LES group. The propensity score was the predicted probability of being in the PES group given the covariate values. Selected covariates included demographics (gender and age), comorbidities at baseline, prior treatment (PCI or CABG), angiographic and procedural characteristics, inotropic agent use, medications administered at discharge, intensive care unit (ICU) duration as well as index and hospitalisation date admission durations (listed in Table 1). We processed the matching using a greedy nearest neighbour algorithm with a calliper of 0.2 times of the propensity score logit’s standard deviation. We assessed the quality of matching using the standardised mean difference (SMD) between groups after matching, and we also considered absolute values lower than 0.1 as having negligible differences. We compared risks of fatal outcomes (i.e., primary composite events, CV death and all-cause death) during follow-ups between the two groups using a Cox proportional hazard model, and we compared the risks of other time-to-event outcomes between the two groups using a Fine and Gray’s sub-distribution hazard model considering all-cause deaths as a competing risk. The study group (second LES vs. PES) was the only one explanatory variable in either the Cox or the Fine and Gray’s models. We performed a pre-specified subgroup analysis on primary composite events to explore whether the beneficial effect of second-generation LESs was inconsistent across different subgroup levels. For patients receiving second-generation LESs before the PSM, we used pairwise log-rank tests to compare the risks of primary composite events among different stents. Finally, we compared the clinical characteristics between the older patients (≥65 years) and younger ones (<65 years) within the PSM cohort, using the *t*- or *χ*^2^-tests. A *P*-value <0.05 was considered statistically significant. We made no multiple testing (multiplicity) adjustments in this study. We performed all statistical analyses using a commercial software (SAS 9.4, SAS Institute, Cary, NC), including procedures of *psmatch* for PSM and *phreg* for survival analyses.

**Table 1:** Baseline characteristics of the patients.

	Before matching			After matching		
Characteristics	PES	2nd LES	SMD	PES	2nd LES	SMD†
Patient number	222	514	–	197	319	–
Age (year)	68.5±12.7	69.0±13.1	-0.04	68.6±12.5	68.7±13.4	-0.01
Gender						
Male	158 (71.2)	375 (73.0)	-0.04	141 (71.6)	232 (72.7)	-0.02
Female	64 (28.8)	139 (27.0)	0.04	56 (28.4)	87 (27.3)	0.02
Prior myocardial infarction	40 (18.0)	60 (11.7)	0.18	32 (16.2)	45 (14.1)	0.06
Prior stroke	31 (14.0)	82 (16.0)	-0.06	28 (14.2)	46 (14.4)	-0.01
Peripheral arterial disease	19 (8.6)	35 (6.8)	0.07	15 (7.6)	23 (7.2)	0.02
Prior PCI	21 (9.5)	34 (6.6)	0.11	16 (8.1)	24 (7.5)	0.02
Prior CABG	11 (5.0)	17 (3.3)	0.09	10 (5.1)	13 (4.1)	0.05
Prior other Comorbidities						
Hypertension	126 (56.8)	277 (53.9)	0.06	113 (57.4)	175 (54.9)	0.05
Diabetes mellitus	105 (47.3)	224 (43.6)	0.07	95 (48.2)	147 (46.1)	0.04
Dyslipidemia	134 (60.4)	359 (69.8)	-0.20	124 (62.9)	212 (66.5)	-0.08
Coronary artery disease	54 (24.3)	103 (20.0)	0.10	46 (23.4)	71 (22.3)	0.03
Heart failure	34 (15.3)	56 (10.9)	0.13	26 (13.2)	42 (13.2)	<0.01
Chronic kidney disease	22 (9.9)	47 (9.1)	0.03	18 (9.1)	34 (10.7)	-0.05
Dialysis	11 (5.0)	28 (5.4)	-0.02	11 (5.6)	20 (6.3)	-0.03
Atrial fibrillation	11 (5.0)	38 (7.4)	-0.10	11 (5.6)	23 (7.2)	-0.07
Gout	14 (6.3)	34 (6.6)	-0.01	13 (6.6)	19 (6.0)	0.02
Chronic obstructive pulmonary disease	22 (9.9)	51 (9.9)	<0.01	19 (9.6)	33 (10.3)	-0.02
Malignancy	16 (7.2)	31 (6.0)	0.05	14 (7.1)	23 (7.2)	<0.01
**Angiographic and Procedural**						
No. of intervened disease vessels						
1	148 (66.7)	337 (65.6)	0.02	132 (67.0)	218 (68.3)	-0.03
2	57 (25.7)	150 (29.2)	-0.08	52 (26.4)	86 (27.0)	-0.01
3	17 (7.7)	27 (5.3)	0.10	13 (6.6)	15 (4.7)	0.08
No. of stents implanted per patient						
1	145 (65.3)	343 (66.7)	-0.03	131 (66.5)	219 (68.7)	-0.05
2	51 (23.0)	113 (22.0)	0.02	44 (22.3)	65 (20.4)	0.05
3	22 (9.9)	36 (7.0)	0.10	19 (9.6)	30 (9.4)	0.01
4 or more	4 (1.8)	22 (4.3)	-0.15	3 (1.5)	5 (1.6)	-0.01
Aspiration catheter used	25 (11.3)	133 (25.9)	-0.38	25 (12.7)	52 (16.3)	-0.09
IABP use	116 (52.3)	313 (60.9)	-0.17	104 (52.8)	174 (54.5)	-0.03
Intubation	85 (38.3)	193 (37.5)	0.02	75 (38.1)	114 (35.7)	0.05
ECMO use	4 (1.8)	41 (8.0)	-0.29	4 (2.0)	7 (2.2)	-0.01
Stay of intensive care unit (days)	9.1±11.1	9.3±10.6	-0.02	9.2±11.5	9.4±11.1	-0.02
Dosage of inotropic medication						
Dopamine (mg×10^3^)	2.2±2.8	2.3±2.9	-0.04	2.0±2.8	2.1±2.9	-0.04
Norepinephrine (mg)	8.6±22.6	13.6±27.9	-0.20	8.3±21.8	9.0±23.2	-0.03
Epinephrine (mg)	5.4±9.3	5.9±9.9	-0.05	5.5±9.4	4.7±8.7	0.09
Medication during index admission						
Aspirin	208 (93.7)	486 (94.6)	-0.04	186 (94.4)	300 (94.0)	0.02
Clopidogrel	218 (98.2)	507 (98.6)	-0.03	193 (98.0)	314 (98.4)	-0.03
Dual antiplatelet	207 (93.2)	484 (94.2)	-0.04	185 (93.9)	299 (93.7)	0.01
B-blocker	136 (61.3)	323 (62.8)	-0.03	125 (63.5)	198 (62.1)	0.03
ACEI/ARB	161 (72.5)	355 (69.1)	0.07	145 (73.6)	228 (71.5)	0.05
Statin	117 (52.7)	333 (64.8)	-0.25	110 (55.8)	195 (61.1)	-0.10
PPI	60 (27.0)	162 (31.5)	-0.10	55 (27.9)	92 (28.8)	-0.02
Calcium channel blocker	67 (30.2)	150 (29.2)	0.02	57 (28.9)	88 (27.6)	0.03
GP IIb/IIIa	6 (2.7)	15 (2.9)	-0.01	6 (3.0)	9 (2.8)	0.01
Index admission duration (day)	17.6±20.8	17.3±19.2	0.01	17.5±20.9	17.8±19.8	-0.01

ACEI, angiotensin-converting enzyme inhibitor; ARB, angiotensin receptor blocker; CABG, coronary artery bypass graft; ECMO, extracorporeal membrane oxygenation; GP: glycoprotein; IABP, intra-aortic balloon pump; LES, limus-eluting stent; PES, paclitaxel-eluting stent; PPI, proton-pump inhibitor; PCI, percutaneous coronary intervention; SMD, standardized mean difference.

Data were presented as frequency and percentage or mean ± standard deviation.

†An absolute SMD of ≤ 0.1 indicates a negligible difference.

## Results

### Patient characteristics

We identified 736 patients with AMI and CS eligible from databases spanning a period from January 2007 to December 2011 for this study. Of those, 222 patients (30.2%) received PES implantation and 514 (69.8%) received second-generation LES implantation. After PSM, 122 patients in the PES group had two counterparts and 75 patients in the PES group had only one counterpart, resulting in a total of 319 patients in the second-generation LES group and 197 patients in the PES group. The average age of the post-matching cohort was 68.6 years (SD = 13.3 years).

Before the PSM, the patients treated with second-generation LESs had a greater proportion of dyslipidaemia, use of aspiration catheter, Intra-aortic balloon pump (IABP), extracorporeal membrane oxygenation (ECMO), statins and a higher norepinephrine dose (absolute SMD >0.1). After the PSM, we found no substantial differences in terms of demographics, comorbidities, angiographic and procedure characteristics, or inotropic agent and medications dosages at discharge between the study groups (absolute SMD <0.1; Table 1). The duration of ICU stay (9.2 days vs. 9.4 days) and the index admission stay (17.5 days vs. 17.8 days) was also similar between the PES and the second-generation LES groups.

### Clinical outcomes

**Fig 1:**
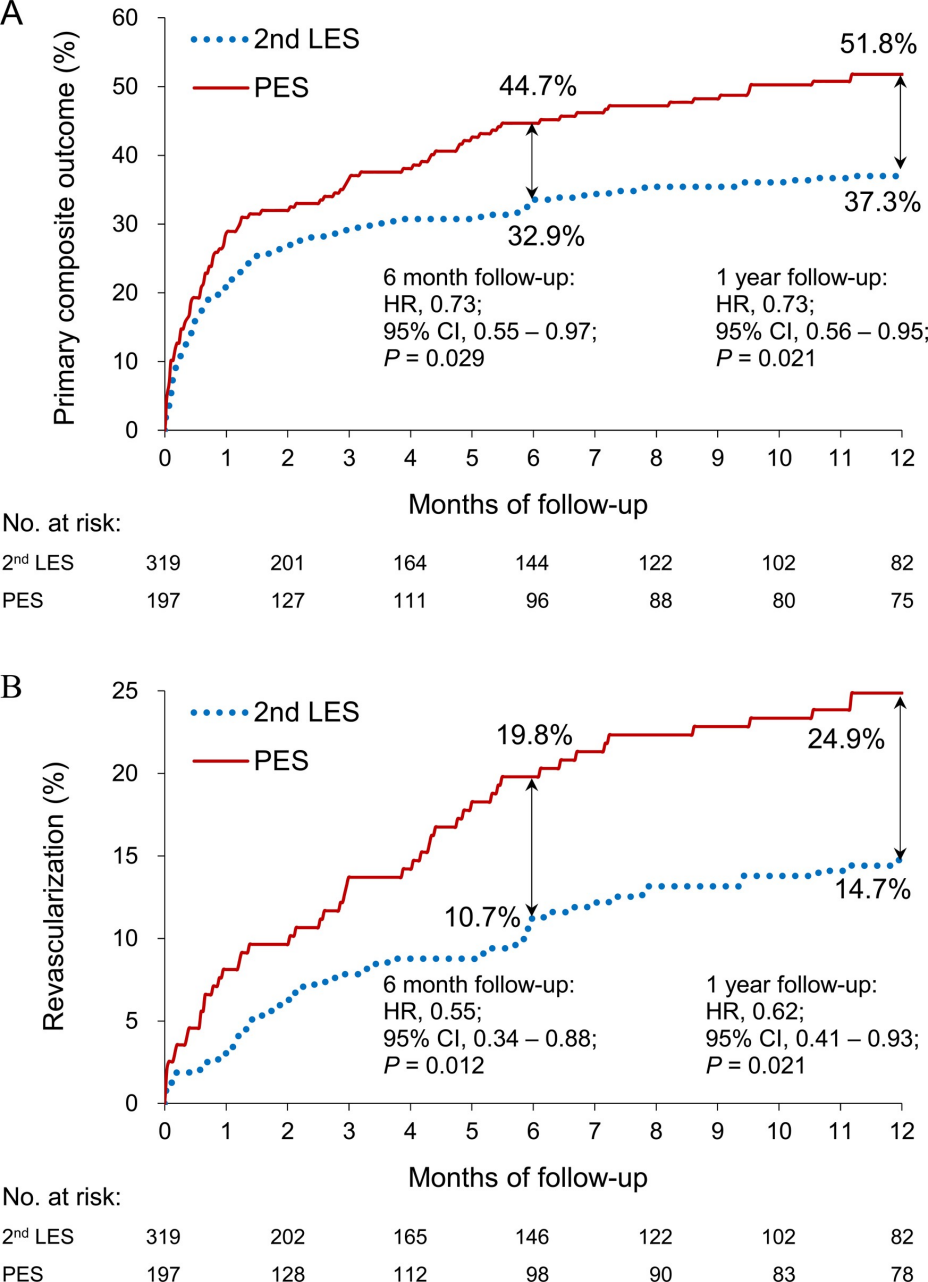
Cumulative event rates of (A) primary composite outcomes and (B) revascularisation during the 6-month and 12-month follow-ups. The primary outcome was a composite of myocardial infarction, coronary revascularisation and cardiovascular death.

The in-hospital mortality rate was 23.4% (46/197) and 21.9% (70/319) in the PESs and second-generation LES groups, respectively. It’s noted that the in-hospital mortality rate did not significantly differ between the two groups (P = 0.710). The proportion of the primary composite outcomes at 6 months was 44.7 and 32.9% in the PES and second-generation LES groups, respectively (Table 2). This difference increased by the 12-month follow-ups (51.8% vs. 37.3%) (Fig 1A). The second-generation LES group was associated with a significantly lower risk of the primary composite outcome at both the 6-month (HR 0.73; 95% CI: 0.55–0.97) and the 12-month follow-ups (HR 0.73; 95% CI: 0.56–0.95).

**Table 2:** Clinical outcome at various follow-up times.

Follow up / Outcome	Number of event (%)		2nd LES *vs*. PES HR (95% CI) †	*P*
	PES (*n* = 197)	2nd LES (*n* = 319)		
**6-month follow up**				
Primary composite events	88 (44.7)	105 (32.9)	0.73 (0.55, 0.97)	0.029
Myocardial infarction	13 (6.6)	11 (3.4)	0.48 (0.21, 1.09)	0.078
Revascularization	39 (19.8)	34 (10.7)	0.55 (0.34, 0.88)	0.012
PCI	33 (16.8)	28 (8.8)	0.55 (0.33, 0.92)	0.022
CABG	7 (3.6)	8 (2.5)	0.67 (0.23, 1.96)	0.469
Cardiovascular death	49 (24.9)	71 (22.3)	0.91 (0.63, 1.31)	0.612
**12-month follow up**				
Primary composite events	102 (51.8)	119 (37.3)	0.73 (0.56, 0.95)	0.021
Myocardial infarction	14 (7.1)	13 (4.1)	0.56 (0.26, 1.24)	0.151
Revascularization	49 (24.9)	47 (14.7)	0.62 (0.41, 0.93)	0.021
PCI	43 (21.8)	41 (12.9)	0.63 (0.41, 0.98)	0.039
CABG	7 (3.6)	8 (2.5)	0.67 (0.23, 1.96)	0.469
Cardiovascular death	52 (26.4)	73 (22.9)	0.90 (0.63, 1.28)	0.540
All cause death	67 (34.0)	106 (33.2)	1.03 (0.76, 1.41)	0.828
Heart failure admission	10 (5.1)	21 (6.6)	1.50 (0.68, 3.31)	0.319
Any CVA	6 (3.0)	8 (2.5)	0.81 (0.28, 2.36)	0.703
Ischemic stroke	5 (2.5)	6 (1.9)	0.73 (0.23, 2.35)	0.596
Hemorrhagic stroke	1 (0.5)	2 (0.6)	1.11 (0.10, 12.93)	0.934
Unspecified stroke	1 (0.5)	1 (0.3)	0.80 (0.04, 14.34)	0.877

HR, hazard ratio; CI, confidence interval; LES, limus-eluting stent; PES, paclitaxel-eluting stent; PCI, percutaneous coronary intervention; CABG, coronary artery bypass graft; CVA, cerebral vascular accident.

† Except for cardiovascular death, all cause death and primary composite events, other outcomes were estimated using Fine and Gray’s subdistribution hazard model which considered all cause death as a competing risk

In terms of the primary outcome components, the patients receiving second-generation LESs had a lower risk of coronary revascularisation compared with those receiving PESs at either the 6-month (HR 0.55; 95% CI: 0.34–0.88) or the 12-month follow-ups (HR 0.62; 95% CI: 0.41–0.93) (Table 2; Fig 1B). We found no group differences regarding other components, including myocardial infarction or CV death.

Regarding secondary outcomes, we found no significant differences in the risks of stroke (HR 0.81; 95% CI: 0.28–2.36), heart failure admission (HR 1.50; 95% CI: 0.68–3.31) or all-cause mortality (HR 1.03; 95% CI: 0.76–1.41) between the second-generation LES and the PES groups (Table 2).

### Subgroup analysis

Among the 514 patients receiving second-generation LESs before the PSM as samples, 140 patients (27.2%) received everolimus-eluting stents (EESs), 316 (61.5%) received zotarolimus-eluting stents (ZESs), 45 (8.8%) received biolimus-eluting stents (BESs) and 13 (2.5%) received mixed types of second-generation LESs. We found no significant differences in the primary composite outcomes of recurrent MI, coronary revascularisation or CV death at the 1-year follow-ups between the different LES subgroups (S2 Fig).

**Fig 2:**
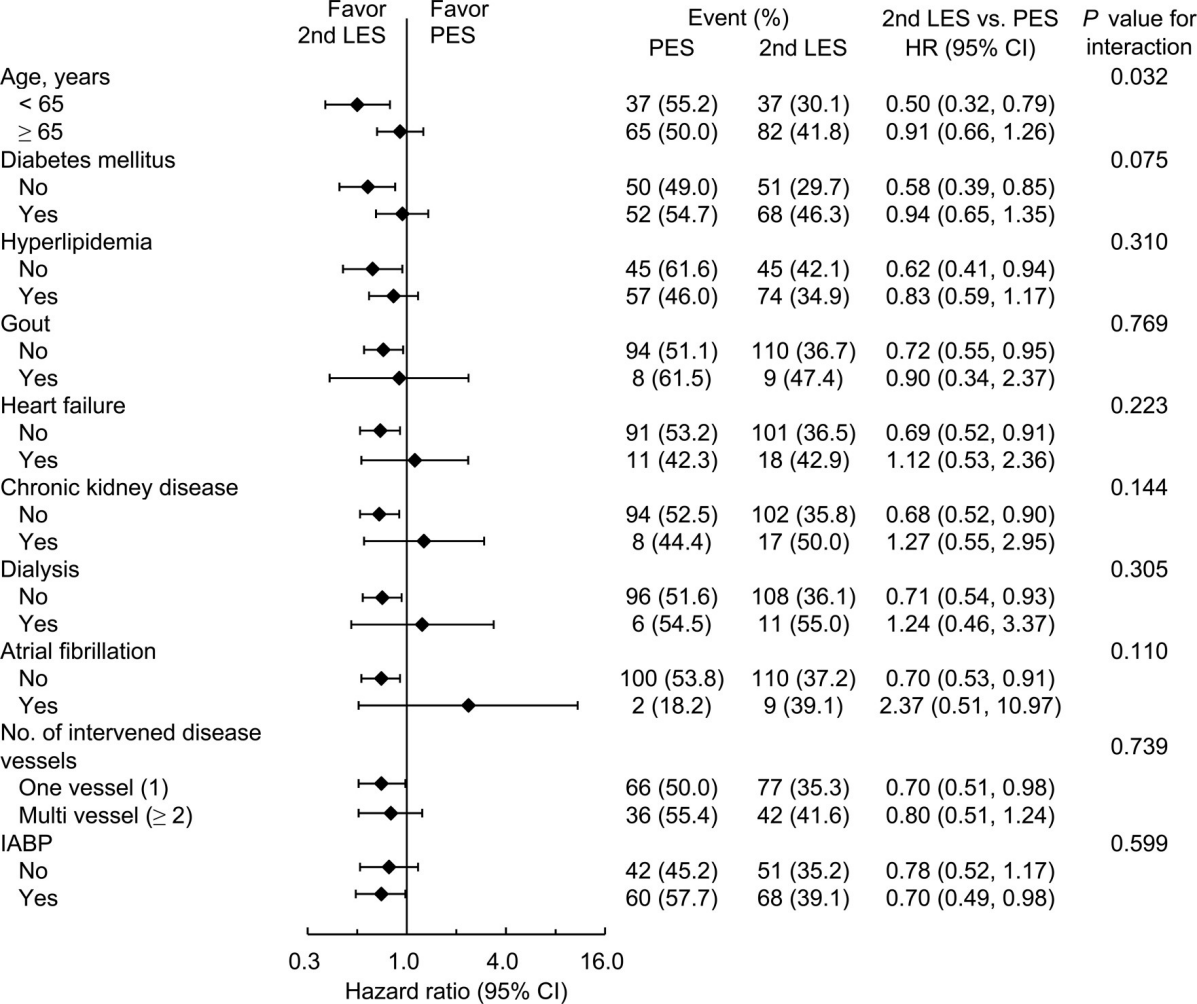
Pre-specified subgroup analysis for primary composite outcomes.

We further analysed the primary composite outcomes at the 12-month follow-up stratified by patient’s characteristics. The beneficial effect of second-generation LESs over PESs was unaffected by the previous history of diabetes mellitus, hyperlipidaemia, heart failure, CKD, dialysis, atrial fibrillation, number of intervened disease vessels or IABP support during the index admission, but was more apparent in the younger population (age <65 years) than in the older population (age ≥65 years) (*P* for interaction = 0.032) (Fig 2).

The baseline comorbidities of the CS-AMI cohort stratified according to being younger or older than 65 years are shown in S2 Table. Compared with patients younger than 65, those who older were so significantly (77.0 years vs. 54.4 years) had more comorbidities such as diabetes (50.9% vs. 40.0%), hypertension (61.3% vs. 46.3%), prior stroke (17.2% vs. 9.5%), atrial fibrillation (8.9% vs. 2.6%), coronary artery disease (28.2% vs. 13.2%) with higher intubation rates (42.6% vs. 26.3%) and longer ICU stay durations (11.0% vs. 6.6%) (S2 Table). The 12-month mortality rates in patients younger and older than 65 years were 15.3% and 44.2%, respectively.

## Discussion

To our knowledge, this is the largest contemporary cohort comparison on clinical outcomes of second-generation LES vs. PES therapy in patients with AMI and CS. The risk of primary composite outcomes was 14.5% lower in the second-generation LES therapy than in the PES therapy at the 12-month follow-ups. Coronary revascularisation was performed less frequently in the second-generation LES group than in the PES group. We found no differences in the rates of MI, ischemic stroke, CV death or all-cause mortality between the two study groups.

Our results suggest a considerably high CV event rate in patients with CS receiving DESs, with 42.8% of the population developing primary composite CV events (5.2% experienced recurrent MI, and nearly one in three (33.5%) patients died by the 12-month follow-ups). Even with these high adverse event rates, we found that in a ‘real-world’ CS-AMI population, second-generation LES implantation is associated with lower revascularisation rates than PES implantation at the 1-year follow-up. The relative benefits of the second-generation LES compared with those of the PES were consistent across the multiple subgroups examined, including those with diabetes mellitus, hyperlipidaemia, heart failure, CKD, dialysis and atrial fibrillation. These findings should help clinicians decide the optimal stent strategy for patients with CS-AMI.

The identity of the most appropriate coronary stent for patients with AMI and CS is still debatable. DESs have been proved to be more efficient than BMSs in reducing repeat revascularisation rates and mortality rates and have also been mentioned by guidelines for treating patients with AMI [22, 23]. Even in the population of CS-complicating AMI, DES implantation can reduce the risk of PCI and death compared to BMS implantation [6]. However, the studies on the types of DESs for this group of patients are limited. The clinical question of whether the safety and efficacy of the second-generation LESs are significantly better than those of the PESs still needs to be addressed in patients with AMI and CS. Studies focusing on patients with stable or unstable angina have proved the benefits of second-generation LESs, such as EESs or ZESs, with reduction in revascularisation compared to PESs at 12-month follow-ups [24, 25]. Possible mechanisms for the decreased revascularisation rate of the second-generation LESs, compared to that of the PESs, may involve the new antiproliferative agents, the relatively thin stent struts with improved, durable, biocompatible polymers that can reduce the stent-mediated endoluminal injury and the endothelialisation time. Another possible explanation is the lower frequency of stent thrombosis of second-generation LESs in patients with AMI [26], although we could not clarify the frequency of stent thrombosis. In the current study, the reduced revascularisation rates and composite outcomes were consistent with those in other studies and further support the clinical benefit of the second-generation LESs versus PESs for this critically, complex patient population (patients with AMI complicated with CS).

The cumulative rates of primary composite outcomes observed at the 1-year follow-ups did not differ significantly across the EES, ZES or BES types in our study. A randomised control trial has demonstrated similar major adverse CV events of MI, revascularisation or death between ZESs and EESs within 12 months [27]. Similarly, the safety and efficacy of ZESs and EESs have also been proved in many different populations including patients with diabetes mellitus [28], Asian populations [29] and also in real-world patients with more than half of the population presenting acute coronary syndromes [30]. Our results are consistent with those in other studies and further demonstrate the comparable clinical outcomes of both EESs and ZESs in patients with AMI complicated with CS.

Interestingly, the beneficial effect of second-generation LESs over PESs was more apparent in the younger population (age <65 years) than in the older population (age ≥65 years). A meta-analysis of EESs versus PESs has suggested a numerical low risk of target lesion failure of EESs in those younger than 65 (HR 0.68; 95% CI: 0.54–0.86) when compared to those older than 65 (HR 0.75; 95% CI: 0.57–0.99) at 3-year follow-ups [11]. For patients with AMI and CS, older age has been linked with higher mortality rates with each additional year consistently conferring approximately 4–5% increased odds of death [31, 32]. In this study, the mean age of those older than 65 was significantly higher than that of those younger than 65 (77.0 years vs. 54.4 years). Patients older than 65 had more comorbidities such as diabetes, hypertension, hyperlipidaemia, coronary artery disease, peripheral artery disease, heart failure, prior MI and prior stroke and had a higher rate of intubation and longer ICU stay durations when compared with those younger than 65. Moreover, the 12-month mortality rate in patients younger than 65 was 15.3%, but it rose up to 44.2% in patients older than 65 years. The possible explanations for the different effect of second-generation LESs over PESs in the different age groups involve baseline comorbidity differences and the higher mortality rates of patients older than 65.

## Study limitations

The strength of our study is its real-world perspective data about clinical outcomes of the second-generation LESs compared with PESs in the high-risk subset of patients with AMI and CS. However, some limitations warrant mention. First, owing to the retrospective nature of our study, the two groups might have had inherent differences. Although we used PSM to balance differences in major characteristics at baseline, LES or PES implantation was selected by the physicians in charge, which may have led to the selection bias. However, before the PSM, the second-generation LES group had a greater proportion of dyslipidaemia, of IABP and ECMO use and higher norepinephrine doses, which suggest an initial higher clinical severity. Even then, we still found a beneficial effect of the second-generation LESs over PESs in our analysis. Second, the NHIRD lacks data on lifestyle factors such as the body mass index, smoking, alcohol consumption and physical activity, all of which are potential confounding factors for each of the outcomes. However, we used chronic obstructive pulmonary disease (COPD) as a proxy variable for smoking, because cigarette smoking is the major factor in COPD and is strongly associated with the prevalence of COPD [33]. Moreover, there is a significant difference of smoking prevalence between men and women (51% in men and 5% in women) in Taiwan [34]. Therefore, by matching the COPD and gender variables between the two study groups, we mitigated the threat of this potential limitation [35]. Third, data did not include the baseline blood pressure, mental status, urine amount or laboratory parameters including serum lactate, troponin and B-type natriuretic peptide levels, which may provide additional information. Finally, we could not clarify the frequency of stent thrombosis due to database limitations.

## Conclusions

Second-generation LESs reduced the risk of major CV events significantly, especially those for coronary revascularisation, compared with PES implantation in patients with AMI and CS undergoing PCI. The beneficial effect was more apparent in the younger population (age <65 years) than in the older population (age ≥65 years). Our results suggest that the second-generation LES is more efficient than PES in patients with CS-complicating AMI.

## Supporting information

S1 Fig Flow chart of participants’ selection. (TIF)

S2 Fig Effect of stent subtypes on the risk of primary composite outcomes during 1-year follow-up using the sample before propensity score matching. (TIF)

S1 Table ICD-9-CM diagnostic codes. (DOCX)

S2 Table The baseline comorbidities of the CS-AMI cohort stratified according to being younger or older than 65 years. (DOCX)
