# Supplementary figures and images for: Clinical outcomes of second-generation limus-eluting stents compared to paclitaxel-eluting stents for acute myocardial infarction with cardiogenic shock
Source: PLoS One. 2019 Apr 3;14(4):e0214417. doi: 10.1371/journal.pone.0214417 (PMC6447233; doi:10.1371/journal.pone.0214417)

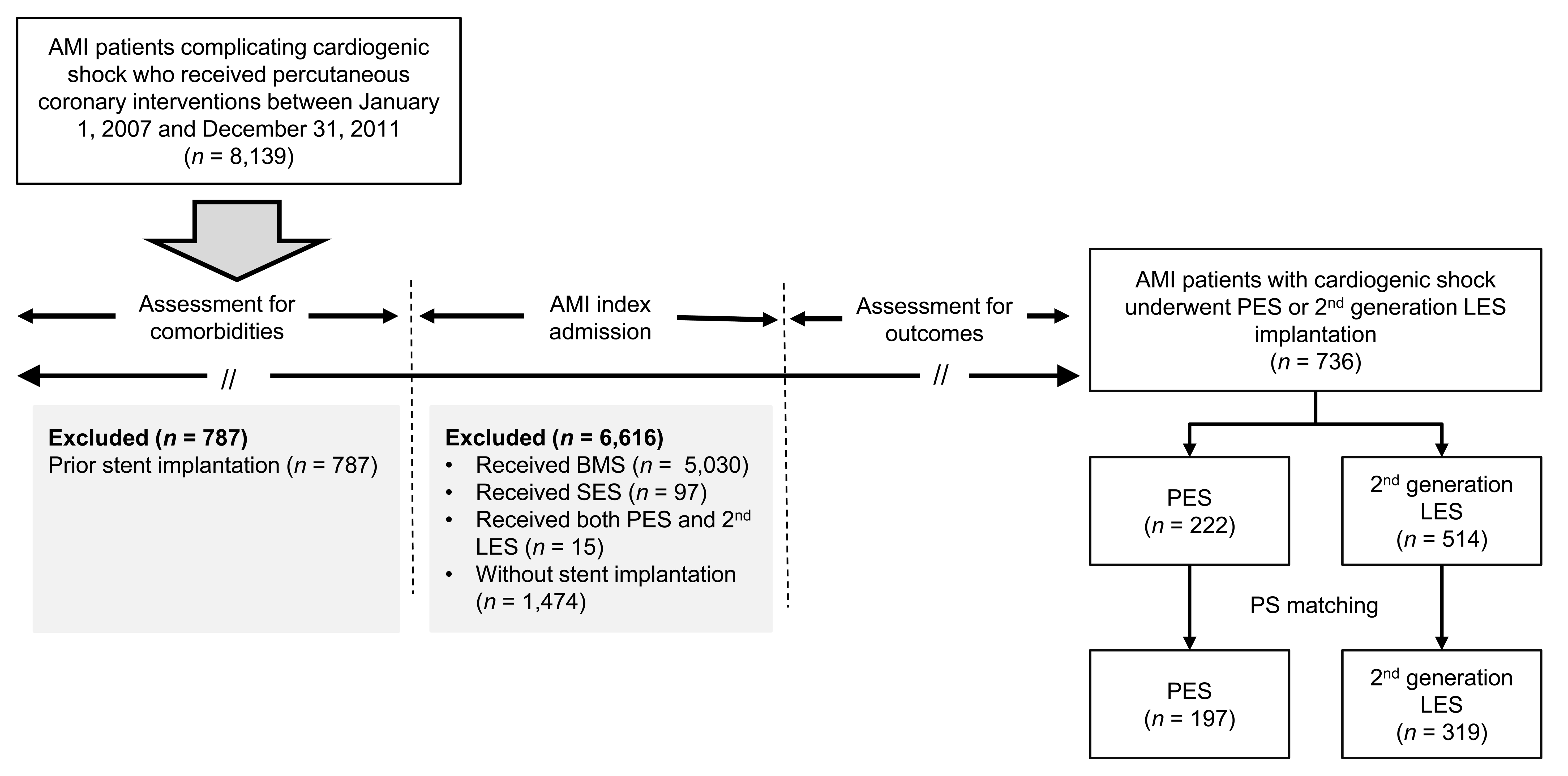

Supplement: S1 Fig — (TIF) [file pone.0214417.s001.tif]

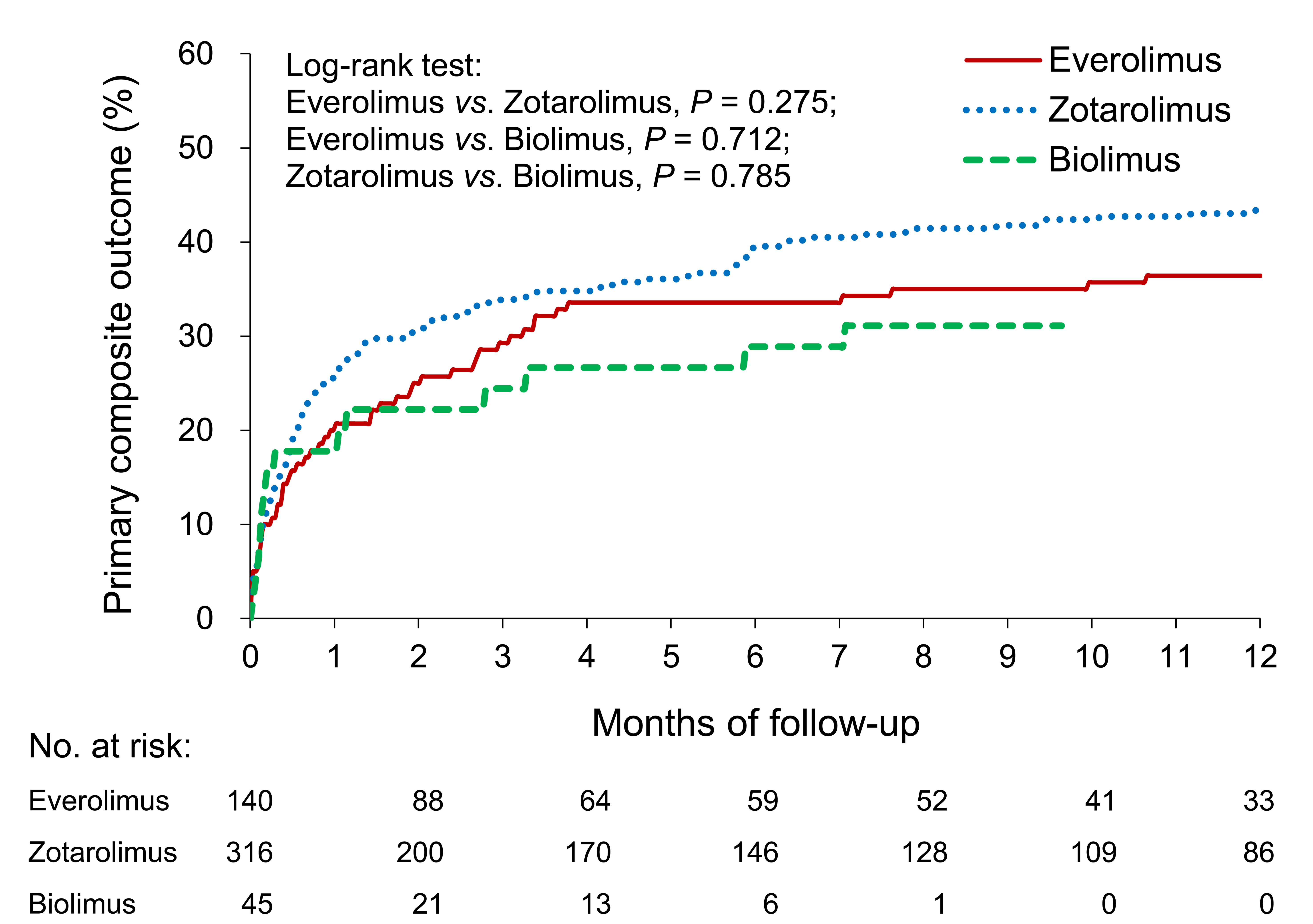

Supplement: S2 Fig — (TIF) [file pone.0214417.s002.tif]
