# Supplementary material for: Clinical outcomes of second-generation limus-eluting stents compared to paclitaxel-eluting stents for acute myocardial infarction with cardiogenic shock
Source: PLoS One. 2019 Apr 3;14(4):e0214417. doi: 10.1371/journal.pone.0214417 (PMC6447233; doi:10.1371/journal.pone.0214417)
Supplement: S1 Table — (DOCX) [file pone.0214417.s003.docx]

**Supplement Table A. ICD-9-CM code used for diagnosis in the current study**

| Variable | ICD-9-CM code |
| --- | --- |
| Acute myocardial infarction | 410.xx |
| Prior myocardial infarction | 410.xx, 412.xx |
| Prior stroke | 430.xx–437.xx |
| Peripheral arterial disease | 440.0x, 440.2x, 440.3x, 440.8x, 440.9x, 443.xx, 444.0x, 444.22, 444.8x, 447.8x, 447.9x |
| Hypertension | 401.xx–405.xx |
| Diabetes mellitus | 250.xx |
| Dyslipidemia | 272.xx |
| Coronary artery disease | 413.xx, 414.0x |
| Heart failure | 428.xx |
| Chronic kidney disease | 580.xx–589.xx, 403.xx–404.xx, 016.0x, 095.4x, 236.9x, 250.4x, 274.1x, 442.1x, 447.3x, 440.1x, 572.4x, 642.1x, 646.2x, 753.1x, 283.11, 403.01, 404.02, 446.21 |
| ESRD (dialysis) | 585.xx (Catastrophic illness card) |
| Atrial fibrillation | 427.31 |
| Gout | 274.xx |
| Chronic obstructive pulmonary disease | 490.xx–496.xx |
| Malignancy | 140.xx–208.xx (Catastrophic illness card) |
| Any CVA | 430.xx–437.xx |
| Ischemic stroke | 433.xx–435.xx |
| Hemorrhagic stroke | 430.xx–432.xx |
| Unspecified stroke | 436.xx–437.xx |

CVA, cerebral vascular accident; ESRD, end stage renal disease; ICD-9-CM, International Classification of Diseases, Ninth Revision, Clinical Modification.
