# Supplementary material for: Clinical outcomes of second-generation limus-eluting stents compared to paclitaxel-eluting stents for acute myocardial infarction with cardiogenic shock
Source: PLoS One. 2019 Apr 3;14(4):e0214417. doi: 10.1371/journal.pone.0214417 (PMC6447233; doi:10.1371/journal.pone.0214417)
Supplement: S2 Table — (DOCX) [file pone.0214417.s004.docx]

**Supplement Table B. Baseline characteristics of the CS-AMI cohort stratified by age (younger or older than 65 years)**

| Characteristics | Age ≥ 65 | Age < 65 | *P* |
| --- | --- | --- | --- |
| Patient number | 326 | 190 | － |
| Age (year) | 77.0±7.0 | 54.4±7.4 | <0.001 |
| Gender |  |  | <0.001 |
| Male | 204 (62.6) | 169 (88.9) |  |
| Female | 122 (37.4) | 21 (11.1) |  |
| Prior myocardial infarction | 57 (17.5) | 20 (10.5) | 0.032 |
| Prior stroke | 56 (17.2) | 18 (9.5) | 0.016 |
| Peripheral arterial disease | 30 (9.2) | 8 (4.2) | 0.036 |
| Prior PCI | 29 (8.9) | 11 (5.8) | 0.203 |
| Prior CABG | 15 (4.6) | 8 (4.2) | 0.836 |
| Prior other Comorbidities |  |  |  |
| Hypertension | 200 (61.3) | 88 (46.3) | 0.001 |
| Diabetes mellitus | 166 (50.9) | 76 (40.0) | 0.017 |
| Dyslipidemia | 200 (61.3) | 136 (71.6) | 0.019 |
| Coronary artery disease | 92 (28.2) | 25 (13.2) | <0.001 |
| Heart failure | 56 (17.2) | 12 (6.3) | <0.001 |
| Chronic kidney disease | 38 (11.7) | 14 (7.4) | 0.119 |
| Dialysis | 22 (6.7) | 9 (4.7) | 0.354 |
| Atrial fibrillation | 29 (8.9) | 5 (2.6) | 0.006 |
| Gout | 22 (6.7) | 10 (5.3) | 0.500 |
| Chronic obstructive pulmonary disease | 42 (12.9) | 10 (5.3) | 0.006 |
| Malignancy | 31 (9.5) | 6 (3.2) | 0.007 |
| **Angiographic and Procedural** |  |  |  |
| No. of intervened disease vessels |  |  | 0.466 |
| 1 | 215 (66.0) | 135 (71.1) |  |
| 2 | 93 (28.5) | 45 (23.7) |  |
| 3 | 18 (5.5) | 10 (5.3) |  |
| No. of stents implanted per patient |  |  | 0.136 |
| 1 | 222 (68.1) | 128 (67.4) |  |
| 2 | 72 (22.1) | 37 (19.5) |  |
| 3 | 25 (7.7) | 24 (12.6) |  |
| 4 or more | 7 (2.1) | 1 (0.5) |  |
| Aspiration catheter used | 33 (10.1) | 44 (23.2) | <0.001 |
| IABP use | 162 (49.7) | 116 (61.1) | 0.013 |
| Intubation | 139 (42.6) | 50 (26.3) | <0.001 |
| ECMO use | 7 (2.1) | 4 (2.1) | 0.975 |
| Stay of intensive care unit (days) | 11.0±12.8 | 6.6±7.2 | <0.001 |
| Dosage of inotropic medication |  |  |  |
| Dopamine (mg×10^3^) | 2.1±2.8 | 2.1±2.9 | 0.839 |
| Norepinephrine (mg) | 10.1±24.5 | 6.4±18.8 | 0.070 |
| Epinephrine (mg) | 5.7±9.2 | 3.7±8.4 | 0.016 |
| Medication during index admission |  |  |  |
| Aspirin | 306 (93.9) | 180 (94.7) | 0.683 |
| Clopidogrel | 320 (98.2) | 187 (98.4) | 0.827 |
| Dual antiplatelet | 304 (93.3) | 180 (94.7) | 0.500 |
| B-blocker | 207 (63.5) | 116 (61.1) | 0.580 |
| ACEI/ARB | 235 (72.1) | 138 (72.6) | 0.894 |
| Statin | 179 (54.9) | 126 (66.3) | 0.011 |
| PPI | 104 (31.9) | 43 (22.6) | 0.024 |
| Calcium channel blocker | 109 (33.4) | 36 (18.9) | <0.001 |
| GP IIb/IIIa | 9 (2.8) | 6 (3.2) | 0.796 |
| Index admission duration (day) | 21.0±22.7 | 12.1±13.2 | <0.001 |

Abbreviations: ACEI, angiotensin-converting enzyme inhibitor; ARB, angiotensin receptor blocker; CS, cardiogenic shock; AMI, acute myocardial infarction; CABG, coronary artery bypass graft; ECMO, extracorporeal membrane oxygenation; GP: glycoprotein; IABP, intra-aortic balloon pump; LES, limus-eluting stent; PES, paclitaxel-eluting stent; PPI, proton-pump inhibitor; PCI: percutaneous coronary intervention.
